# Lifelong Bilingualism Functions as an Alternative Intervention for Cognitive Reserve Against Alzheimer's Disease

**DOI:** 10.3389/fpsyt.2021.696015

**Published:** 2021-07-22

**Authors:** Haiqing Liu, Longhuo Wu

**Affiliations:** ^1^Department of Foreign Languages, School of Humanities and Social Sciences, Gannan Medical University, Ganzhou, China; ^2^Department of Pharmacy, Gannan Medical University, Ganzhou, China

**Keywords:** bilingualism, cognitive decline, cognitive reserve, dementia, Alzheimer's disease

## Abstract

Bilingualism has been reported to significantly delay the onset of dementia and plays an important role in the management of Alzheimer's disease (AD), a condition inducing impairment in the brain network and cognitive decline. Cognitive reserve is associated with the adaptive maintenance of neural functions by protecting against neuropathology. Bilingualism acts as a beneficial environmental factor contributing to cognitive reserve, although some potential confounding variables still need further elucidation. In this article, the relationship between bilingualism and cognitive reserve is discussed, interpreting the advantage of bilingualism in protecting against cognitive decline. In addition, the possible brain and biochemical mechanisms, supporting the advantageous effects of bilingualism in delaying the onset of dementia, involved in bilingualism are reviewed. Effectively, bilingualism can be considered as a pharmacological intervention with no side effects. However, the investigation of the pharmacological parameters of bilingualism is still at an early stage.

## Introduction

The rapid growth in the older population poses significant challenges to individuals and society, particularly in the field of protection against age-associated cognitive decline. Cognitive decline caused by aging has been reviewed comprehensively (1). Clinically, aging is the greatest risk factor for developing Alzheimer's disease (AD). AD has been considered the most prevalent form of dementia; it causes damage to the brain network and consciousness, subsequently inducing cognitive decline and dementia (2). The prevalence of AD was conservatively estimated at 5% for people over 60 in 2007 (3), and the number of people diagnosed with AD worldwide may reach 100 million by 2050 (4). The risk of AD can be affected by interactions between environmental variables and individual genes.

Cognitive decline caused by aging might be associated with changes in the neuroanatomical (5) and neurophysiological (6) characteristics and in the functional connectivity of the brain regions (7). Cognitive reserve generally refers to the capability of individuals to cope adaptively with neuropathology in order to maintain cognitive functioning using neural resources. Cognitive reserve suggests that the efficiency, capacity, and flexibility of cognition are modulated by life experience, regardless of quantitative brain measures (8). Life experience includes educational attainment, occupational attainment, leisure and social activities, and bilingualism/multilingualism.

Bilingualism is regularly referred to as using two (or more) languages: a native language (L1) and a second language (L2). More than 50% of individuals worldwide speak two or more languages. The different effects of bilingualism and monolingualism on cognitive abilities were identified by Saer in 1923. The neurocognitive profiles of bilingualism are sensitive to L2-associated variables, such as age of acquisition, exposure, and proficiency. Increasing evidence reports that lifelong bilingualism contributes to cognitive reserve and delays the onset of cognitive decline due to AD by an average of 4–5 years (9–11). In bilingual patients with mild cognitive impairment (MCI), the clinical onset of cognitive complaints has been shown to occur 7.4 years later than for non-bilingual patients. (12). Lifelong bilingualism produces a more efficient application of brain resources, enabling patients to maintain cognitive functions acting against neuropathology. This effect has been shown to be most apparent when the L2 is used proficiently or if the L2 is acquired early in life (13). However, the underlying mechanisms of bilingualism in protecting against cognitive decline/dementia, particularly in MCI or AD, are still unclear. In this article, we will discuss the relationship between bilingualism and cognitive reserve.

## Bilingualism Contributes to Cognitive Reserve

**Figure 1:**
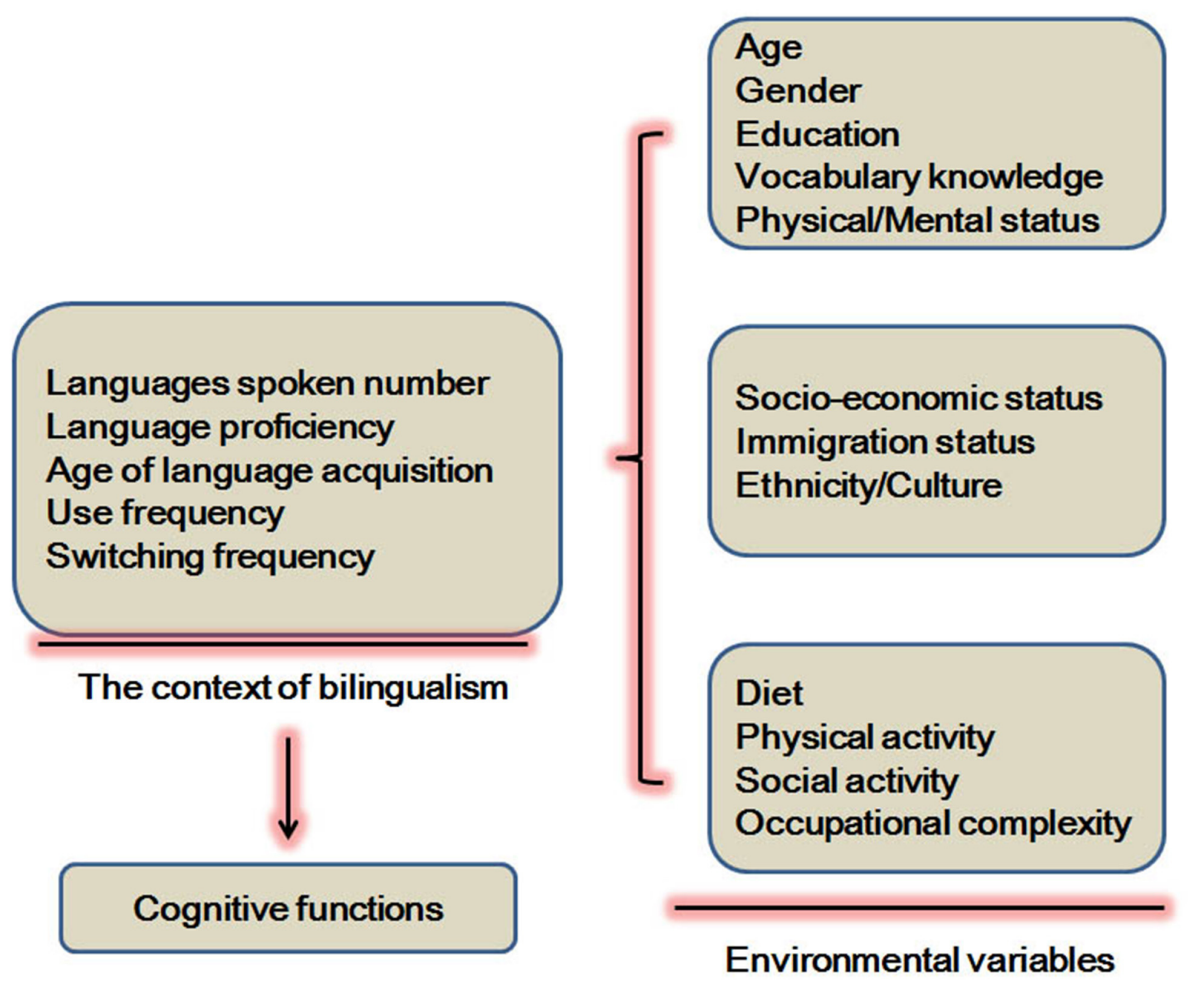
Certain environmental variables involved in the effects of bilingualism on cognitive functions.

Different environmental variables (Figure 1) have been shown to influence cognitive reserve. These variables are education, occupation, socioeconomic status, early life linguistic ability, and cognitive activities (14). For example, bilingual education and L2 maintenance benefit cognitive reserve in patients with AD (15). However, the effects of years of higher education are not significantly related to cognitive reserve in MCI (12). In the United States, first-generation Mexican–American immigrants were studied to investigate potential confounding variables such as immigrant status and cultural heterogeneity. Bilingualism demonstrates modest benefits in the cognitive screening performance of older individuals (16). In addition, AD onset and the first clinical visit in bilingual speakers of Cantonese and Mandarin is found to occur at an older age when compared to monolingual Mandarin speakers and Cantonese bilingual speakers (17).

### Effects of Bilingualism on Verbal Activity

Early bilinguals exhibit slower lexical retrieval of words with low frequency and smaller word production, particularly in category or semantic fluency (18), with a possible mechanism activating a more diffuse network than would be true for monolinguals (19, 20). However, late bilinguals obtain better scores than monolinguals (20). A study shows that frequency lag and competition account may influence the verbal activity of bilingual people. In terms of the frequency lag, bilinguals speak each word in both languages less often than monolinguals, resulting in reduced accessibility of words in both languages. Bilinguals score below monolinguals on vocabulary in one of their languages. However, their total vocabulary from the two languages is dramatically more extensive than that of monolinguals (21). In inflecting novel words, patients with probable or prodromal AD appear to weaken the impact of phonological analogy only at the more severe disease stages, associated with the impairment of phonological working memory (22).

A loss of language skills is often seen in patients with dementia and it also becomes one of the most challenging issues of their condition. In dementia, difficulty in word finding is the first clinically presenting abnormality. Then, reduced verbal fluency, naming, and comprehension are observed in sequential order (23). In addition, a reduced rate of verbal learning acquisition has been related to major neurocognitive disorder (MaND), which can help to differentiate MaND from mild neurocognitive disorder (MiND) and from no neurocognitive disorder (NoND). Reduced word recall differentiates MiND from NoND (24). However, for bilinguals with dementia, this difficulty is often rather complex due to the influence of cross-language interference, leading to lack of control in fluency maintenance for L1 and L2 (25). A survey study on individuals with MCI who speak Spanish as an L1 found a consistent preference for Spanish but not for their L2, English. Even more concerning, when they speak English, certain Spanish words and phrases are often included (26). Bilinguals with AD may experience a gradual decrease in the fluency of their two languages, regressing to strongly preferring the dominant language, that is, the first one learned (26).

### Effects of Bilingualism on Non-verbal Activity

Language use produces a mediating activity on the age-related decline of executive functions in bilinguals. Interestingly, bilinguals consistently exhibit better executive control and enhanced performance in non-verbal tasks that require similar skills, such as anticipation, attention, conflict monitoring, interference suppression, response inhibition, and task switching (27). A study reports that late bilinguals who have similar use of two languages show an advantage in conflict resolution, and early bilinguals exhibit improved monitoring processes (28). Bilinguals with a higher rate of language switching in daily life exhibit decreased switch costs relating to the characterization of a given task compared to monolinguals. Furthermore, bilinguals suffer less interference from cross languages relating to conflict resolution and obtain better scores on executive control tasks associated with inhibitory control (29). Early bilinguals show fewer switching costs than late bilinguals (30). Late bilingualism results in more cognitive advantages with stronger inhibition mechanisms, as indicated by a better performance in the Simon task (30). Consistently, bilingualism specifically benefits the costs of resolving cognitive conflict, independent of the difference in raw reaction time (31).

Older bilingual individuals have demonstrated better task-switching performance in a color shape task compared with monolinguals. Mechanically, the left lateral frontal cortex and cingulate cortex in bilinguals have been revealed to show lower activation as detected by functional MRI (fMRI), indicating more efficiency in executive functions (32). Another study reports that poor performance on tests for verbal memory in patients with aphasia is postulated to be the result of language impairment. It has been demonstrated that bilingualism with concomitant diagnoses of psychiatric diseases may complicate the interpretation of cognitive testing. Hopefully, a combination of imaging findings over time and symptoms evolution may be of benefit in differentiating primary progressive aphasia from other pathological conditions (33).

Both languages used by bilinguals are activated when the target language is being used. Therefore, bilinguals constantly cope with competition in selecting lexical forms between the two languages by inhibiting the non-target language. Activation of two or more languages may continuously train the executive functions, leading to a higher engagement of cognitive control mechanisms in bilingual individuals (34). This process indicates the existence of a control mechanism preventing interference. fMRI analysis indicates that early bilinguals and monolinguals show increased activation in the brain areas responsible for classic language choices, such as the left inferior frontal cortex. The main difference in brain area activation depends on the age of L2 acquisition and the L2 proficiency (35).

In addition, early bilinguals exhibited lower performance in a picture-naming test, while late bilinguals achieved better scores (20). Similarly, bilinguals exhibited better performance on a grammar judgment task and a non-verbal visual search task (36). A study reports that picture naming and comprehension (spoken word-to-picture matching) in L1 in bilingual patients with semantic dementia are impaired, and their corresponding levels in L2 are much lower. Even more concerning is that some patients exhibit a loss of ability in L2 or in the third language (L3). These severely impaired performances can be observed in early and late bilinguals. Less proficient and later-acquired L2 bilingual individuals are more vulnerable to neuropathology in degenerative diseases, such as AD (37).

Patients with amnestic MCI are clinically diagnosed by their memory deficit with or without damage in other cognitive domains. Clinically, single-domain amnestic MCI exhibits memory problems alone; multiple-domain cases involve damage in memory, executive functions, languages, or visuospatial ability. It has been demonstrated that only patients with single-domain amnestic MCI are related to diagnosis at a later age in bilinguals than in monolinguals. The protective advantage of bilingualism in multiple-domain amnestic MCI has not been observed (38). Bilinguals with probable AD have been shown to preserve more long-term memory by increasing the activities of the cognitive domain affected by AD than monolinguals with probable AD (39, 40). Higher levels of cognitive reserve or resilience are positively associated with functionality in posterior areas and efficient memory (41). A study reports that bilingualism may delay the clinical symptoms of early-stage AD by promoting executive control rather than memory and by protecting regions of the brain (42).

Bilingualism can be defined by early L2 acquisition, high level of L2 proficiency, balanced use of two languages, and high frequency in switching languages. Higher levels of bilingualism are demonstrated to be positively associated with greater executive control. However, active bilinguals do not score better in long-term memory tasks (43), although the brain activities maintained by bilingualism are theoretically higher in the posterior areas and produce more efficient memory. However, results from event-related potential (ERP) measuring the working memory performance show that no significant differences in accuracy and reaction time were observed. Larger P2 and P3b amplitudes were found in bilinguals, indicating more resources available in cognitive processing (44). A systemic review suggests that bilingualism is not associated with neuropsychological test performance in longitudinal studies in patients with MCI. However, these contradictory findings might be due to the difference in methodology relating to sample collection, protocols, and the inconsistent evaluation of tests (21). Increased executive functions in bilinguals have been suggested as a reason as to why bilinguals score below monolinguals in vocabulary and other language-format cognitive tests using a single language. Moreover, the delayed onset of dementia as a consequence of bilingualism could not be confirmed by additional rigorous incidence research (45, 46).

Collectively, bilingualism tunes brain structures and networks important for executive control, resulting in neuroprotective effects.

## The Mechanisms of Bilingualism in Protecting Against Cognitive Decline

The onset of AD can be delayed by sustained stimulating activities, including higher educational and occupational achievement, leisure and social activities, and bilingualism/multilingualism. However, these variables are difficult to evaluate or quantify. No study can exclude all the confounding variables relating to bilingualism. However, different variables relating to bilingualism may have a crucial impact on cognitive reserve. A single variable could not take into account the possible interactions between variables. Interestingly, matching groups by using a multivariate method rather than by a univariate method has a significant effect on the results. Older healthy bilinguals exhibit higher axial diffusivity in the left superior longitudinal fasciculus than monolinguals do when matching on seven variables, including verbal and spatial intelligence quotient (IQ), age, education, Trial-Making Test (TMT), Mini Mental State Examination (MMSE), and gender, and their interactions. These results indicate a later cognitive decline in bilinguals, and show that L2 experience benefits the neural reserve (47).

It is necessary to acknowledge that these environmental variables could not prevent neuropathology or other brain damage. Instead, they may alleviate the effects of neuropathology on clinical symptoms. Specifically, bilingualism does not slow down the development of atrophy in the mesial temporal regions in dementia type AD. Instead, bilinguals with probable AD exhibit more significant amounts of hippocampal and mesiotemporal atrophy than monolinguals do (48, 49). Interestingly, increased neuronal activity may effectively postpone the pathological changes of AD. The explanation could be brain plasticity in bilinguals and the possibility that bilingualism could result in dramatic resistance to cognitive decline. Conceptually, bilingualism can be considered as an enriching exercise contributing to neuroplasticity.

### The Possible Brain Mechanisms of the Cognitive Protective Effects of Bilingualism

A positive relationship between neuropathology and cognitive decline has also been documented. The interaction between bilingualism and cognition can be bidirectional (31). Individuals with a higher cognitive reserve may withstand more neuropathology and delay their cognitive decline. In turn, neuroplastic changes are implicated in the activity of speaking more than one language. Also, increased functional connectivity may compensate for disease-related cognitive decline and may be a factor in bilingualism (50). MRI for brain structure in healthy aging suggests that cognitive reserve is positively associated with the volumes of gray and white matter in the frontal and temporoparietal cortices (51). A decreased volume of gray matter in the prefrontal cortex detected by anatomical brain imaging is linked to cognitive impairment (5). In patients with MCI, active bilinguals exhibit lower white matter integrity in the fornix but higher integrity in the parahippocampal cingulum and uncinate fasciculus than is the case for passive bilinguals, indicating a differential pattern of white matter disintegration (52).

**Figure 2:**
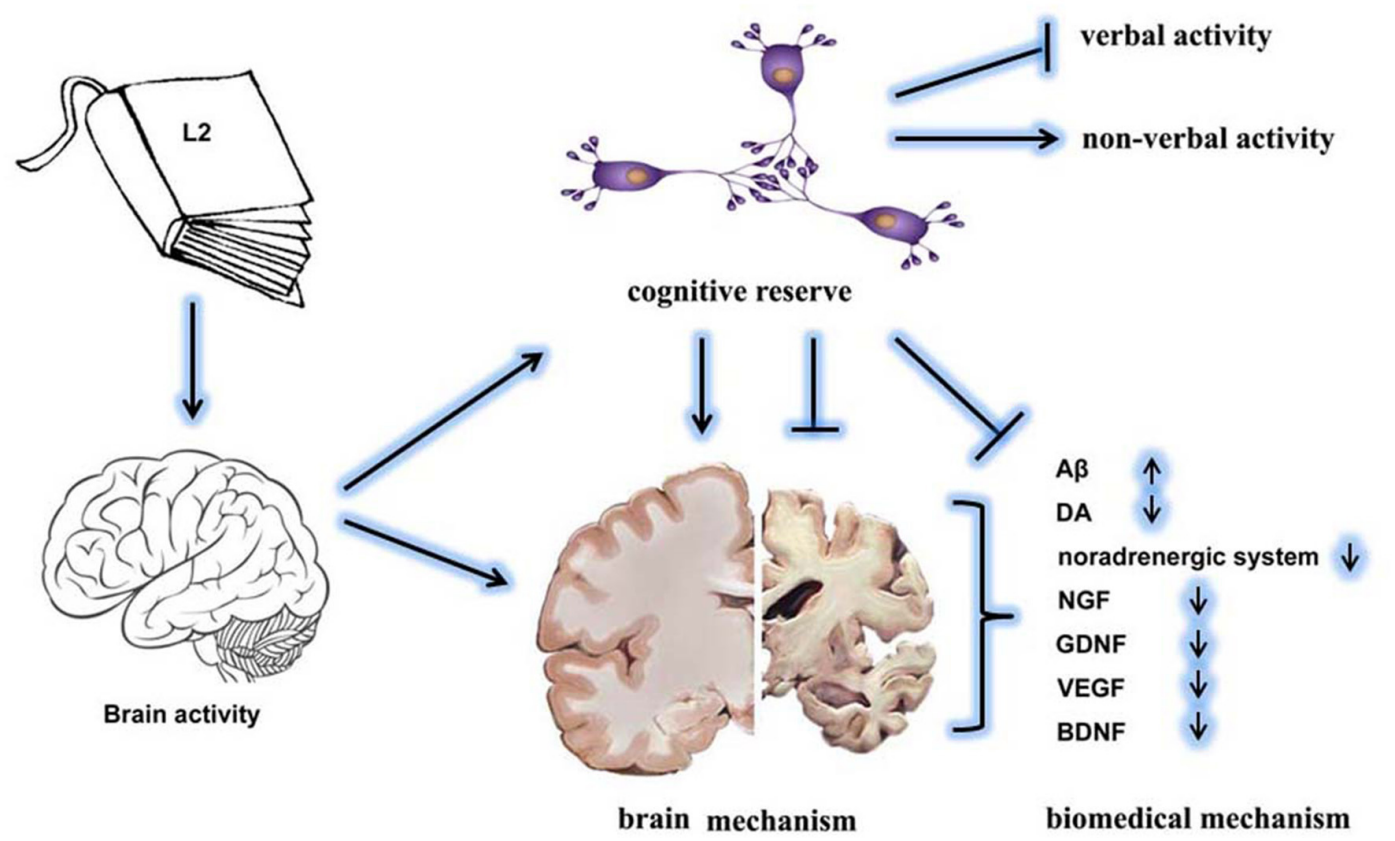
The effects of bilingualism on delaying the onset of dementia. The L2 language exposure increases brain activity and cognitive reserve. Specifically, bilingualism increases non-verbal activity but decreases verbal activity. The underlying mechanisms might include brain mechanisms and biochemical mechanisms. Bilingualism-enhanced cognitive reserve effectively protects the brain structure and maintains the homeostasis of biochemical factors.

Bilingualism has been developed as a strategy to delay the onset of AD-related dementia (11, 53). Mechanically, bilingualism contributes to cognitive reserve by two brain mechanisms: neural reserve and neural compensation (Figure 2). Results from neuroanatomic research indicate that bilingualism increases the volume of gray matter and the integrity of white matter (54). Consistently, bilingualism protects against the age-related decrease of gray-matter volume in the left temporal pole (55). The density of gray matter in the left inferior parietal region has been found to be positively correlated with the proficiency in L2 (56). A study reports that multilinguals with early-stage AD have a thicker cortex than that of monolinguals in the frontal and related areas, which are responsible for performance in episodic memory tasks, indicating memory compensation through increased executive control abilities (57). Bilingualism shows improved maintenance in white matter connectivity between the frontal and posterior areas than is seen in monolinguals (58). In addition, lifelong bilinguals have higher neocortical gray and white matter lobar volumes and gray-matter structure in the temporal lobe than is seen in monolinguals, indicating the adaptive effects of bilingualism on enhancing neuronal connections among brain areas (59). It has been demonstrated that structural changes in the inferior frontal gyrus in bilinguals are associated with L2 training. Also, the thickness of the inferior frontal gyrus in bilinguals is reported to be affected by the age of L2 acquisition (60). Aging bilinguals are also found to have consistently greater temporal pole volume, which is related to proficiency in L2 (55). Recently, brain structure changes induced by bilingualism have been reviewed, indicating they include the left inferior parietal lobule, the anterior cingulate cortex, and the subcortical structures. These brain areas are the regions that form the executive control network, thereby explaining the cognitive advantage of bilingualism in performing executive control tasks (54). Neural compensation demonstrates the need to maintain cognitive functions in the presence of brain atrophy by increased utilization of the brain network. This compensation improves neural reserve, as shown by the increased size in specific brain areas in overcoming pathology and neurological insult (8). These findings suggest that bilinguals with probable AD show similar levels of cognitive reserve and memory and cognition functions, while showing similar levels of temporal neuropathology to those of monolinguals (48).

The neurofunctional mechanisms underlying each language in bilinguals with high proficiency are more convergent than those for bilinguals with low proficiency (61). Recently, brain structures in patients with AD correlated with the less proficient or more proficient language performance were investigated using the Multilingual Naming Test (MINT). The MINT performance of AD patients with more proficient language is correlated with the cortical thickness of the entorhinal cortex and middle temporal gyrus. In contrast, the performance arising from the less proficient language was associated with thickness of the left caudal anterior cingulate cortex, a region implicated in error monitoring and task switching (62).

However, meta-analysis reveals that bilinguals do not show significant differences in reducing the risk of dementia development compared with monolinguals. No difference in disease severity at dementia diagnosis was observed between bilinguals and monolinguals, although the bilinguals were older (63). The current neuroimaging techniques for interpreting the effects of bilingualism on the volume of gray and white matter are still at a developmental stage. Many aspects of correlations between behavioral data and variables influencing comparisons between bilinguals and monolinguals in tasks are still unclear (46).

### The Possible Biochemical Mechanisms of Cognitive Protective Effects of Bilingualism

The accumulation of amyloid β (Aβ) or neurofibrillary tangles indicates cognitive decline, increasing the possibility of a diagnosis of dementia type AD (64) (Figure 2). Another study had similar results indicating that high cognitive reserve (high education, high literacy, and no *APOE* e4 allele) indicates less or no association with the plasma Aβ-42/40 level and rate of decline (65). In addition, functions of the dopamine (DA) system are involved in the regulation of age-related reduction in episodic memory, executive control, and perceptual speed. Age-related DA losses decrease the signal-to-noise ratio in the functional neuron networks (66) (Figure 2).

Repeated activation of the noradrenergic system over a lifetime has a neuroprotective effect in mediating the activity of the environmental enrichment on the brain and cognitive reserve against AD, comprehensively reviewed in an earlier study (67) (Figure 2). It is presumed that activation of the noradrenergic signaling pathway could be a possible mechanism linked to the effects of bilingualism on the delayed onset of dementia (68). In terms of the effects on the neural system, many neural factors, such as nerve growth factor (NGF), glial-derived neurotrophic factor (GDNF), vascular endothelial growth factor (VEGF), and brain-derived neurotropic factor (BDNF), are hypothesized as being involved in the protection afforded by bilingualism against cognitive decline (69) (Figure 2).

The possible biochemical mechanisms of bilingualism in protecting against AD type of dementia have been investigated. Brain metabolism and connectivity have been measured using fluorodeoxyglucose (FDG)-positron emission tomography (PET), and bilingualism shows a more severe pattern of cerebral hypometabolism in several posterior brain regions and hypermetabolism in the orbitofrontal, inferior frontal, and cingulate cortex (40). Connectivity between the posterior cingulate, subcortical regions/structures and anterior cingulate has been identified in bilinguals with probable AD, indicating a compensation effect on brain neurodegeneration (40, 70). This notion is supported by metabolic connectivity analysis, demonstrating enhanced connectivity in the executive control and the default mode networks in bilinguals with probable AD (40). Neuropathological research shows that early bilingualism is linked to a lower level of the cerebrospinal fluid AD biomarker—tau and a lower prevalence of preclinical/prodromal AD, contributing to executive and visual-spatial functions and cognitive reserve (20).

## Perspectives

Increasing evidence demonstrates that learning more than one language effectively improves mental performance beyond the field of language. Bilingualism seems to have an impact on cognitive functioning at all ages (71). Importantly, L2 acquisition might be a factor promoting compensatory reserve and delaying cognitive decline in dementia (72). Individuals intensively learning L2 over 3 months show a significant increase in hippocampus volume and cortical thickness in the left middle frontal gyrus, inferior frontal gyrus, and superior temporal gyrus (73). Structural changes in native speakers of English who had been learning Swiss German for 5 months indicate a close association between the left inferior frontal gyrus and proficiency in L2 (74). The possible mechanisms responsible for why L2 learning promotes cognitive reserve have been discussed (75). Bilingualism has been viewed as a powerful, safe, and cheap therapy, with no side effects (76). The degree of bilingualism proficiency, in our opinion, can be considered as “the dose” needed to treat dementia. However, compared to most pharmacological interventions, the dose-response curve of bilingualism is still unclear, although a threshold of 5 h/week is recommended for learning L2 (77). It should be noted that only those bilinguals with lifelong exposure to and use of both languages will obtain the maximum benefits (55). This is because these individuals take advantage of efficient or alternative neural networks, and dementia is one of the main neuropathological symptoms associated with age-associated diseases, such as AD.

Once the clinical symptoms of dementia appear, the neuropathology of bilinguals probably deteriorates more rapidly than that of monolinguals (78, 79). Bilingualism promotes cognitive reserve with improved executive control and increases the neural reserve with improved left frontal and related areas and connections. This may result in delaying the onset of dementia because of compensatory effects, but it does not alter its pathology (49, 80). In other words, bilingualism delays the symptoms of dementia but not the disease itself. However, as dementia develops and begins to affect the frontal language control, bilinguals are more vulnerable to asymmetrical language impairment, resulting in increased reversion to the L1 because of the increasing cost of suppressing the L1. Bilinguals with dementia have more advanced temporal neuropathology than do monolinguals at comparable clinical stages (80). Also, the number of behavioral symptoms is vast, and this feature may be yet another sign of brain degeneration. Specifically, bilinguals/multilinguals show poor performance on the Boston naming test and have more delusional ideas (81). Dementia has been shown to affect language skills and conversational performance. The L2 performance can be much lower than the L1 performance. However, this difference disappears as the disease develops (82). It is possible that high-proficiency bilinguals use their L2 more implicitly by engaging the procedural memory system. In contrast, low-proficiency bilinguals seem to engage the declarative memory system, which is more vulnerable during AD development (83).

It has been shown that bilingualism produces a better cognitive outcome after stroke, as indicated by the higher percentage of intact cognitive functions in bilinguals than in monolinguals. However, bilingualism does not seem to be a predictor of post-stroke cognitive impairment. There are no differences between bilinguals and monolinguals in the vascular risk or the stroke age (84). Another study shows that higher frequency in using bilingualism is closely related to better performance in inhibitory control and set-shifting and higher gray-matter volume in the inferior frontal gyrus, protecting against Huntington's disease, which is characterized by progressive motor, cognitive, and neuropsychiatric alternations. These factors might be associated with increased metabolism in multiple frontotemporal regions, including the dorsal anterior cingulate cortex, the anterior insula, the ventromedial orbital prefrontal cortex, and the inferior frontal gyrus (85).

There is no difference between multilingual and bilingual individuals in the prevalence of dementia, although multilingual patients with dementia tend to have a 2-year delay in the onset of symptoms when compared with bilinguals (86). Also, no difference was found in the onset of dementia between bilinguals and monolinguals in non-immigrant samples (87). In addition, no association was observed between bilinguals and monolinguals in the diagnosis of dementia (88). Bilingualism does not protect against cognitive decline, nor does it enhance the executive functions as measured by MMSE scores; the protective effects of bilingualism over time may be due to the use of language and not to bilingualism in itself (89). Surprisingly, the advantage of bilingualism in delaying dementia is questionable. An explanation for that situation could include a bias against the publication of non-significant results from some research studies with low to medium statistical levels, a bias on selecting the involved patients, or a bias on the reporting of the age of onset of dementia (90).

In addition, one study shows that no significant difference emerged from prospective studies of bilingualism and monolingualism in their level of protection from cognitive decline or dementia. Although this study also identifies retrospective studies on the positive effects of bilingualism in delaying the onset of cognitive decline, the authors are not prepared to accept this concept due to the vulnerability of retrospective studies to being confounded by the educational and cultural differences among the participants (91). It has been argued that these prospective studies are incomplete due to potential inaccuracies in the establishment of bilingualism (92). There is also a discrepancy insofar as bilingualism could be excluded as a categorical variable as it is confounded by the type and proficiency of bilingualism (43, 93). The inconsistent pieces of evidence have been analyzed, and a plea for methodological innovation has been developed (94).

## Limitations

The investigation of bilingualism in relation to dementia is controversial (45). Many plausible explanations are provided. First, the definition of bilingualism is inconsistent. Possibly, the most important feature influencing research results is the lack of an objective determination of L2 proficiency. In many research studies, L2 levels are determined using subjective self-reports, leading to inaccurate assessments (95). Second, the varieties and subtypes of bilingual individuals are different. There are simultaneous or sequential bilinguals corresponding to the early or late age of acquisition. Third, there are other important confounding variables, including education, immigrant status, family structure, acculturation, and native language (96). Finally, there can be emotional, social, and cultural problems relating to the two languages (97). In this article, we have discussed the beneficial role of lifelong bilingualism in promoting cognitive reserve against AD. Research data has demonstrated that bilingualism may delay the onset of cognitive decline for 4–5 years in patients with AD (11) and 7.4 years in patients with MCI (12).

High cognitive reserve has a positive influence on cognitive performance. However, high cognitive reserve does not modify the rate of change in cognition (98). Many studies suggest that cognitive reserve produces different effects according to APOE genotype, mainly demonstrating that the protective effects of cognitive reserve against dementia are stronger in healthy subjects with the ε4 allele (99). One study demonstrated that cognitive reserve is a risk factor for progression from MCI to AD dementia in the ε4+ group, while no statistically significant effects were observed in the ε4- group (100).

In non-clinical older adults, cognitive reserve is positively associated with cognitive functions, but not with the rate of change: that is, cognitive reserve does not predict the rate of cognitive decline (101). However, in individuals who are cognitively impaired or diagnosed with AD dementia, higher cognitive reserve is related to accelerated decline (78, 79). This discrepancy can be attributed, at least in part, to the difference in the proportion of individuals in each cohort who progress to MCI or dementia (78). Another study explains that the difference might be associated with different levels of educational attainment across cohorts, different measures of variables (102), or methodological limitations (103).

The application of bilingualism in the prevention of AD in adulthood reveals multiple obstacles, such as motivation, cost, and low frequency of use. In addition, the difference between adult status and childhood in learning and acquiring new languages introduces structural and functional changes.

## Conclusions

The high prevalence of dementia, combined with the continuing growth in the older population, poses enormous challenges to the economy and society. Learning or speaking L2 will do no damage. It could significantly reduce the potential costs for individuals and the social economy. More than half of the population in the world is bilingual. However, it is hardly plausible to propose that these individuals are protected from dementia. Lifelong bilingualism is associated with more efficiency in using brain resources, protecting cognitive functioning against neuropathology. A better understanding of how bilingualism affects and delays the onset of dementia could potentially encourage individuals to investigate how to increase the possibility of living independently for a longer period of time. It is noteworthy that the organizational structures of different languages in the world and the cognitive mechanisms engaged by them may result in differential patterns in the advantages of bilingualism as a protection against dementia. The influence of bilingualism on the development of AD and other diseases causing cognitive decline should be further investigated. The focus of future studies should move beyond determining the delayed onset of clinical AD symptoms, challenging current theoretical frameworks, and establishing a profile of empirical data that could inform clinical practice. The debate on the contribution of bilingualism to cognition remains wide open.

## Author Contributions

LW provided the concept for this article. HL wrote, revised, and finalized the article. Both authors have approved the final article.

## Conflict of Interest

The authors declare that the research was conducted in the absence of any commercial or financial relationships that could be construed as a potential conflict of interest.
